# Plant responses to UV-B radiation: signaling, acclimation and stress tolerance

**DOI:** 10.1007/s44154-022-00076-9

**Published:** 2022-12-05

**Authors:** Zhiren Chen, Yuan Dong, Xi Huang

**Affiliations:** State Key Laboratory of Cellular Stress Biology, School of Life Sciences, Faculty of Medicine and Life Sciences, Xiamen University, Xiamen, 361102 China

**Keywords:** UV-B stress, Photoreceptor, UVR8, Flavonoid, Transcription factor

## Abstract

Ultraviolet-B (UV-B) light is an intrinsic part of sunlight that reaches the earth’s surface, and affects plant survival and adaptation. How plants respond to UV-B light is regulated by the wavelength, intensity and duration of UV-B radiation, and is also regulated by photosynthetically active radiation perceived by phytochrome and cryptochrome photoreceptors. Non-damaging UV-B light promotes plant photomorphogenesis and UV-B acclimation which enhances plant tolerance against UV-B stress. However, high-level UV-B radiation induces DNA damage, generates reactive oxygen species (ROS) and impairs photosynthesis. Plants have evolved efficient mechanisms to utilize informational UV-B signal, and protect themselves from UV-B stress. UV RESISTANCE LOCUS8 (UVR8) is a conserved plant-specific UV-B photoreceptor. It interacts with CONSTITUTIVELY PHOTOMORPHOGENIC1 (COP1) to initiate UV-B-specific light signaling and regulate UV-B responsive gene expression. A set of transcription factors such as ELONGATED HYPOCOTYL5 (HY5) function downstream of the UVR8-COP1 module to promote seedling de-etiolation for photomorphogenic development and biosynthesis of sunscreen flavonoids for UV-B stress tolerance. In addition to UVR8 signaling pathways, plants subjected to damaging UV-B radiation initiate stress protection and repair mechanisms through UVR8-independent pathways. In this review, we summarize the emerging mechanisms underlying UV-B stress acclimation and protection in plants, primarily revealed in the model plant *Arabidopsis thaliana*.

## Introduction

Light provides plants with the energy source needed for photosynthesis and acts as an important environmental cue to regulate plant survival and development. However, light can also function as an abiotic stress factor for plants [e.g., high light, ultraviolet-B (UV-B) radiation] (Demarsy et al. 2018; Kami et al. 2010; Shi and Liu 2021; Yadav et al. 2020). UV-B (280–315 nm) light is an intrinsic part of sunlight that reaches the earth’s surface. Plants are inevitably exposed to UV-B light of varied levels throughout their life cycle. Their physiological responses to UV-B light are regulated by the wavelength, intensity and duration of UV-B irradiation (Jenkins 2009), and are also affected by photosynthetically active radiation perceived by phytochrome and cryptochrome photoreceptors (Rai et al. 2020; Tissot and Ulm 2020). Plants perceive UV-B light through the plant-specific UV-B photoreceptor UV RESISTANCE LOCUS8 (UVR8) (Kliebenstein et al. 2002; Rizzini et al. 2011), which elicits specific light signaling for photomorphogenic and acclimatory responses, including seedling de-etiolation, leaf and root development, phototropism, flowering and the biosynthesis of phenylpropanoid derivatives (Arongaus et al. 2018; Dotto et al. 2018; Favory et al. 2009; Kim et al. 1998; Kliebenstein et al. 2002; Wargent et al. 2009; Yadav et al. 2020; Yang et al. 2020). Accumulated in the UV-B acclimation, the phenylpropanoid derivatives flavonols serve as sunscreen to contribute to UV-B stress tolerance. However, high-level UV-B irradiation provokes UV-B stress which damages plant growth and impairs their development. Plants have evolved efficient mechanisms to utilize UV-B light signal and protect themselves from UV-B stress. In this review, we summarize the current understanding on UV-B-induced stress pathways and protective mechanisms in plants, particularly focusing on recent progress revealed in *Arabidopsis thaliana* about UVR8-dependent and -independent pathways that contribute to UV-B stress tolerance.

## UV-B stress-induced damage

While most of UV‐B radiation is absorbed by the atmospheric ozone layer, approximately 5% of the solar UV-B radiation reaches the earth’s surface (Roy 2017). UV-B radiation is potentially harmful to living organisms exposed to sunlight. Excessive UV-B radiation not only causes skin cancers in humans (D’Orazio et al. 2013; Santiago et al. 2021), but also leads to cell death in plants physiologically featured with wilting, yellowing or bleaching of leaves (Jenkins 2009). UV-B stress can induce damage directly at the DNA level by producing cyclobutene pyrimidine dimers (CPDs), and pyrimidine (6–4) pyrimidinone photoproducts (6–4 PPs) (Britt 1995; Molinier et al. 2008; Quaite et al. 1994; Sinha and Hader 2002; Takahashi et al. 2011). DNA lesions produced by UV-B stress affect both DNA replication and transcription, thereby inhibiting plant development and metabolism. Meanwhile, UV-B stress increases the levels of reactive oxygen species (ROS), a group of molecules derived from molecular oxygen (O_2_), which induces oxidative stress and oxidizes DNA, RNA, proteins, lipids and many small molecules in plant cells (D’Orazio et al. 2013; Demarsy et al. 2018; Hideg et al. 2013; Hollosy 2002; Mittler et al. 2022). In the process of photosynthesis, UV-B radiation directly damages photosynthetic machinery, primarily photosystem II (PSII), by degrading the PSII proteins D1 and D2 (Hollosy 2002; Takahashi et al. 2010). In addition to photosystem damage, UV-B radiation reduces Rubisco activity and chlorophyll contents, resulting in low photosynthetic capacity in plants (Frohnmeyer and Staiger 2003; Sztatelman et al. 2015).

## Exposure limitation, protection and repair mechanisms against UV-B stress

Plant survival under UV-B radiation is achieved by the combined action of exposure limitation, protection, and repair mechanisms. The best strategy to cope with light stress is to simply avoid it. Chloroplasts can change their positions to minimize the absorption of high light, thereby avoiding PSII damage (Wada et al. 2003). To further limit UV-B-induced damage to PSII, plants adopt various strategies to maintain the balance between repair and PSII damage in response to high UV‐B light. PSII damage is repaired efficiently by a PSII repair mechanism involving the disassembly, degradation, and neo-synthesis of the D1 subunit and the reassembly of PSII (Takahashi and Badger 2011). UV-B-absorbing sunscreen phenylpropanoids, including flavonoids, and polyphenols, accumulate in epidermal cells to protect plants from potentially damaging UV-B radiation (Favory et al. 2009; Jenkins 2017; Kliebenstein et al. 2002; Podolec and Ulm 2018; Stracke et al. 2010). *Arabidopsis* mutants deficient in flavonoid and hydroxycinnamic acid biosynthesis (*transparent testa 4* [*tt4*] and *tt5*, defective in flavonoid biosynthesis; *uv-sensitive* [*uvs*], defective in kaempferol biosynthesis; *ferulic acid hydroxylase 1* [*fah1*], defective in sinapate ester biosynthesis) suffer from increased sensitivity to UV-B radiation (Tanaka et al. 2002).

UV-B-induced DNA damage (CPDs and 6–4 PPs) can be repaired efficiently by photolyases. Pyrimidine dimers can be repaired by nucleotide excision repair (NER), or bypassed by replicative polymerases (Britt 2004). The expression of the CPD photolyase (*PHR*) gene is induced by UV-B light dependent on UVR8 signaling pathway, and is also induced by blue and UV-A light (Li et al. 2015). Many key regulators involved in DNA damage repair were initially isolated via genetic screen. UV-B-induced DNA damage repair is impaired in the *Arabidopsis* mutants *uv resistance 1* (*uvr1*) (Britt et al. 1993), *uvr2* (Jiang et al. 1997; Landry et al. 1997), *uvr3* (Jiang et al. 1997; Nakajima et al. 1998), and *uv hypersensitive 1* (*uvh1*) (Harlow et al. 1994). By contrast, *uv-b insensitive 1* (*uvi1*) mutant showed enhanced DNA repair activity (Tanaka et al. 2002).

UV-B-induced ROS are scavenged by enzymatic and non-enzymatic antioxidants (ROS-scavenging systems) in plants (Mittler et al. 2022). Small antioxidants (e.g., ascorbic acid, carotenoids, flavonoids, glutathione, proline, α-tocopherol) and scavenging enzymes such as ascorbate peroxidase (APX), catalase (CAT), glutathione peroxidase (GPX), peroxidase (PRX), superoxide dismutase (SOD), thioredoxin-dependent peroxidase (TPX) and other antioxidants play important roles in ROS scavenging (Mittler 2017; Waszczak et al. 2018). *Arabidopsis* mutants deficient in ascorbic acid biosynthesis (*vitamin c defective 1* [*vtc1*] and *vtc2*) and in tocopherol cyclase activity (*vitamin e deficient 1* [*vte1*]) exhibit oxidative damage in response to light-stimulated stress (Gao and Zhang 2008; Porfirova et al. 2002; Yao et al. 2015). UV-B radiation enhances APX activity in *Arabidopsis* (Rao et al. 1996), and promotes the accumulation of small antioxidant flavonoids in leaf epidermal cells to enhance plant tolerance to UV-B stress (Hsieh and Huang 2007).

## UV-B stress tolerance mediated by UVR8 signaling

UVR8 is a plant-specific UV-B photoreceptor that was evolutionarily originated in green algae (Han et al. 2019; Rizzini et al. 2011). *UVR8* was originally isolated as a UV-resistance gene through genetic screen for the mutants hypersensitive to UV-B stress in *Arabidopsis* (Kliebenstein et al. 2002). According to the defects found in *uvr8* mutants, which showed longer hypocotyl, less flavonoid accumulation, and more damage under UV-B radiation than wild-type plants, UVR8 has been identified as a key positive regulator in UV-B-induced photomorphogenic development and stress acclimation (Brown et al. 2005; Jenkins 2014; Rizzini et al. 2011; Tilbrook et al. 2013). Another role of UVR8 has been proposed in maintaining photosynthetic efficiency though via unknown molecular mechanism (Davey et al. 2012). Further, the requirement of UVR8 orthologs for UV-B stress tolerance has been demonstrated by genetic studies in both higher and lower plants, such as *Solanum lycopersicum*, *Marchantia polymorpha*, and *Chlamydomonas reinhardtii* (Allorent et al. 2016; Kondou et al. 2019; Li et al. 2018; Liu et al. 2020; Tilbrook et al. 2016).

*UVR8* encodes a 440-amino acid protein with two functional domains, a seven‐bladed β-propeller core domain and a C-terminal C27 domain (Christie et al. 2012; Rizzini et al. 2011; Wu et al. 2012; Yin et al. 2015). The molecular basis of UVR8 as a UV-B photoreceptor has been illuminated by structural and biochemical studies. In the absence of UV-B light, UVR8 forms a homodimer that is stabilized by the salt bridge through electrostatic interactions between the charged amino acids (primarily Arg-286 with Asp-107/Asp-96 and Arg-338 with Asp-44). Though UVR8 contains no external cofactor, Trp-285 and Trp-233, which are located in the homodimeric interface, serve as the UV-B chromophore (Christie et al. 2012; Wu et al. 2012). Upon UV-B absorption, UVR8 is photoactivated via a structural switch from a dimer to a monomer (Christie et al. 2012; Rizzini et al. 2011; Wu et al. 2012). At the experimental removal of UV-B radiation, UVR8 can revert to its inactive dimer form on its own in vitro, while this process is dramatically accelerated in vivo by REPRESSOR OF UV-B PHOTOMORPHOGENESIS 1 (RUP1) and its homolog RUP2 (Gruber et al. 2010; Heijde and Ulm 2013; Heilmann and Jenkins 2013; Podolec et al. 2021a; Wang et al. 2022). These two UV-B inducible WD40 proteins act downstream of UVR8 in a negative feedback loop, to balance UV-B-induced development and stress defense (Gruber et al. 2010).

At the photoreceptor level, the molecular mechanisms by which plants protect themselves from UV-B stress are associated with the regulation of UVR8 activity, conformation and subcellular localization. It has been revealed that the constitutive or enhanced photoreceptor activity of UVR8 is achieved by point mutations of the key residues responsible for UV-B perception and dimer stabilization, such as UVR8^W285A^, UVR8^R338A^, UVR8^G101S^, and UVR8^D96N,D107N^ (Heijde et al. 2013; Huang et al. 2013, 2014; Podolec et al. 2021b). These UVR8 variants lead to enhanced UV-B-induced photomorphogenesis that promotes UV-B stress tolerance. In addition to RUP1 and RUP2 that directly mediate UVR8 redimerization and inactivation via protein–protein interaction (Gruber et al. 2010; Heijde and Ulm 2013), phytochromes and cryptochromes indirectly promote UVR8 inactivation by upregulating *RUP1* and *RUP2* expression, and thus negatively regulate UVR8 signaling. Reciprocally, BLUE-LIGHT INHIBITOR OF CRYPTOCHROMES1 (BIC1) and BIC2 which inhibit cryptochrome dimerization to repress their activation are upregulated by UV-B signaling (Tissot and Ulm 2020). This finding has provided a molecular insight that photoreceptors of visible and UV-B light co-regulate UV-B stress tolerance through signaling interplay under natural light environment (Rai et al. 2020; Tissot and Ulm 2020). At the subcellular level, UV-B light promotes the nuclear accumulation of UVR8, leading to UV-B responsive gene expression (Kaiserli and Jenkins 2007). Further, glucocorticoid receptor (GR)-based conditional localization system has been employed to clarify that UVR8 signaling predominantly occurs in the nucleus to mediate UV-B-induced photomorphogenesis and stress acclimation (Qian et al. 2016; Yin et al. 2016). The nuclear UVR8 monomers are derived from dimer-to-monomer switch within the nucleus and through the cytoplasm-to-nucleus translocation (Qian et al. 2016).

To mediate UV-B light signal transduction, monomerized UVR8 interacts with the E3 ubiquitin ligase CONSTITUTIVELY PHOTOMORPHOGENIC1 (COP1), through the β-propeller domain and the C-terminal Val-Pro (VP) motif of UVR8 and the C-terminal WD40 domain of COP1 (Favory et al. 2009; Rizzini et al. 2011; Yin et al. 2015). This interaction sequesters COP1 from CULLIN4-DAMAGED DNA BINDING PROTEIN 1 (CUL4-DDB1)-based E3 ubiquitin ligase complex, which serves to destabilize the central photomorphogenesis-promoting transcription factor ELONGATED HYPOCOTYL 5 (HY5) in darkness (Chen et al. 2010; Huang et al. 2013; Osterlund et al. 2000), and also enables photoactivated UVR8 to compete with HY5 for COP1 binding (Favory et al. 2009; Lau et al. 2019), allowing HY5 accumulation for UV-B- induced photomorphogenesis and stress acclimation. The mechanism of binding COP1 via VP motif is well conserved among plant photoreceptors and their signaling components in the regulation of COP1 E3 activity (Favory et al. 2009; Lau et al. 2019; Ponnu et al. 2019; Rizzini et al. 2011; Wang and Lin 2019). With the assistance of COP1, UVR8 accumulates in the nucleus to initiate downstream UV‐B signaling pathways (Favory et al. 2009; Kaiserli and Jenkins 2007; Qian et al. 2016; Rizzini et al. 2011; Wu et al. 2012; Yin et al. 2016). As UVR8 accumulates in the nucleus preferably in its monomeric form, this process is negatively regulated by RUP1 and RUP2 due to their repression of UVR8 monomer levels (Qian et al. 2016).

## UVR8 dependent UV-B responsive gene expression

Early transcriptomic analyses have illuminated that each of UVR8, HY5 and COP1 is essential for genome-wide UV-B responsive gene expression to govern UV-B-induced photomorphogenesis and stress acclimation (Brown et al. 2005; Favory et al. 2009; Ulm et al. 2004). UVR8 harbors no typical DNA binding domain, but its ability to associate with chromatin has been proposed and experimentally examined based on its sequence similarity with REGULATOR OF CHROMATIN CONDENSATION 1 (RCC1), a guanine nucleotide exchange factor (GEF) for the Ran GTPase (Brown et al. 2005; Cloix and Jenkins 2008; Kliebenstein et al. 2002). It is of note that whether the direct binding of UVR8 to chromatin occurs in vivo still remains controversial (Binkert et al. 2016). Although exactly how UVR8 or the UVR8-COP1 complex shapes UV-B-regulated gene expression is not well understood, it has been elucidated that the nucleus-localized UVR8 governs UV-B responsive transcriptional networks in concert with multiple transcription factors (TFs), involving the modulation of the stability and activity of transcription factors (Podolec et al. 2021a; Qian et al. 2020).

In *Arabidopsis*, the basic leucine zipper (bZIP) transcription factor HY5 plays a central role in UV-B light signaling, along with HY5-HOMOLOG (HYH) (Ulm et al. 2004). The UV-B-induced expression of *HY5* depends on UVR8 and COP1 (Brown et al. 2005; Favory et al. 2009; Oravecz et al. 2006). In response to UV-B radiation, HY5 not only associates with the promoters of its downstream target genes involved in UV-B signaling and DNA damage repair, such as *RUP1*, *RUP2*, *UVR2* and *UVR3* (Job et al. 2022), but also associates with its own promoter in positive feedback regulation (Binkert et al. 2014). A set of genes encoding R2R3-MYB transcription factors are also directly activated by HY5, including *MYB11*, *MYB12* and *MYB111* (Stracke et al. 2007). Together with HY5, these MYB transcription factors are responsible for the expression of flavonoid biosynthetic genes, such as *CHALCONE SYNTHASE* (*CHS*), *CHALCONE ISOMERASE* (*CHI*), and *FLAVONOL SYNTHASE1* (*FLS1*), so as to play positive roles in UV-B stress tolerance in *Arabidopsis* (Stracke et al. 2010).

*Arabidopsis* B-box (BBX) family transcription factors play important roles in UV-B signaling and stress tolerance through functional connection with HY5. UV-B light induces *BBX24/SALT TOLERANCE* (*STO*) expression and stabilizes its protein accumulation. BBX24 negatively regulates UV-B-induced photomorphogenesis by interacting with both COP1 and HY5, and repressing HY5 activity (Jiang et al. 2012). *BBX31* expression is induced by UV-B light in a UVR8-, COP1-, and HY5-dependent manner. Though as a negative regulator of photomorphogenesis under white light, BBX31 is a positive regulator of photomorphogenesis and stress protection under UV-B radiation, by regulating gene expression involved in photoprotection and DNA repair relied on HY5 (Yadav et al. 2019). BBX20, BBX21 and BBX22 act as HY5 coactivators to allow the sustained expression of genes involved in flavonoid biosynthesis (Podolec et al. 2022). HY5 regulates the UV-B-mediated induction of *BBX11* by directly binding to its promoter. BBX11 reciprocally regulates *HY5* mRNA and HY5 protein levels (Job et al. 2022). FAR-RED ELONGATED HYPOCOTYL3 (FHY3) is another transcription factor that positively regulates UV-B-induced photomorphogenesis and stress acclimation. By binding to distinct regulatory elements within the *COP1* promoter, FHY3 directly activates *COP1* while HY5 promotes *COP1* expression via a positive feedback loop. FHY3 and HY5 physically interact with each other and this interaction is diminished by UV-B radiation (Huang et al. 2012).

At the posttranslational level, HY5 protein stability is regulated by RUP1/RUP2 and COP1 (Huang et al. 2013; Ren et al. 2019). Beyond their roles in UVR8 redimerization and inactivation, RUP1 and RUP2 act as UV-B inducible substrate receptors of CUL4-DDB1-based E3 ligase to mediate the degradation of HY5. To alleviate their repression of HY5, COP1 interacts with RUP1 and RUP2 for ubiquitination and degradation, in order to indirectly stabilize HY5 (Ren et al. 2019). Another group of transcription factors that regulate gene expression for elongation growth, PHYTOCHROME INTERACTING FACTOR 4 (PIF4) and PIF5, are subjected to UV-B-induced and UVR8-dependent degradation via the ubiquitin–proteasome system (Sharma et al. 2019; Tavridou et al. 2020).

To mediate UV-B signaling for stress acclimation, UVR8 directly interacts with several transcription factors and modulate their activity in gene expression regulation. *MYB13* is a UV-B inducible gene predominantly expressed in cotyledons, and its expression is positively regulated by the nuclear localization of UVR8 following UV-B exposure. As a member of R2R3-MYB transcription factor family, MYB13 directly binds to the promoters of *CHS*, *CHI* and *FLS* and activates their expression. Photoactivated UVR8 interacts with MYB13 and enhances its binding to these target promoters, so as to promote flavonoid accumulation and UV-B tolerance (Qian et al. 2020). The direct interaction of UVR8 with WRKY DNA-BINDING PROTEIN 36 (WRKY36) and BRI1-EMS-SUPPRESSOR 1 (BES1)/BES1-INTERACTING MYC-LIKE 1 (BIM1) impairs their DNA binding activity, in order to alleviate the inhibition of UV-B responsive gene expression and repress brassinosteroid-related gene expression respectively, and thereby positively regulates UV-B-induced photomorphogenesis and stress acclimation (Liang et al. 2018; Yang et al. 2018). On the other hand, brassinosteroid (BR) signaling inhibits UV-B stress responses in plants by limiting flavonoid biosynthesis. BR-activated BES1 represses the expression of *MYB11*, *MYB12*, and *MYB111* in a BR-enhanced manner. However, exposure to UV-B stress downregulates *BES1* expression, and releases its inhibition of these *MYB* genes, thus promoting flavonoid accumulation and enhancing UV-B stress protection in a UVR8-independent manner (Liang et al. 2020). Therefore, BR signaling and BES1 mediate the tradeoff between plant growth and stress defense in UVR8-dependent and -independent pathways according to UV-B radiation levels.

## UVR8-independent UV-B stress tolerance

When UVR8-mediated acclimation is insufficient to resist UV-B stress, the mitogen-activated protein kinase (MAPK) signaling pathway is activated as a complementary strategy for stress tolerance (Besteiro et al. 2011). MAPK signaling cascades can be specifically triggered by UV-B-induced DNA damage, other than DNA replication stress (Gonzalez Besteiro and Ulm 2013). MAPK PHOSPHATASE 1 (MKP1) interacts with and dephosphorylates its downstream MPK3 and MPK6 in *Arabidopsis* (Bartels et al. 2009). MKP1 knockout results in hypersensitivity to acute UV-B stress but normal UV-B acclimation, with reduced inactivation of MPK3 and MPK6. However, loss-of-function mutation of either MPK3 or MPK6 leads to elevated UV-B stress tolerance and partially suppresses the UV-B hypersensitivity of *mkp1*. Though UVR8 is crucial in prior UV-B acclimation for further stress tolerance, it does not obviously contribute to acute UV-B stress. Therefore, MKP1 and its substrates MPK3 and MPK6 antagonistically regulate UV-B stress tolerance in a UVR8-independent manner (Besteiro et al. 2011).

In parallel to MAPK signaling pathway, two paralogous phosphoinositide 3-kinase-like kinases (PIKKs), ATAXIA TELANGIECTASIA MUTATED (ATM) and ATM-AND RAD3-RELATED (ATR) mediate plant tolerance against double-strand breaks (DSBs) and DNA replication stress respectively (Culligan et al. 2006; Sancar et al. 2004). These two parallel pathways coordinate spatiotemporal regulation of UV-B stress responses, as MKP1 plays a predominant role in the shoots while ATR in the roots (Gonzalez Besteiro and Ulm 2013).

## Other pathways to mediate plant stress responses

**Fig. 1:**
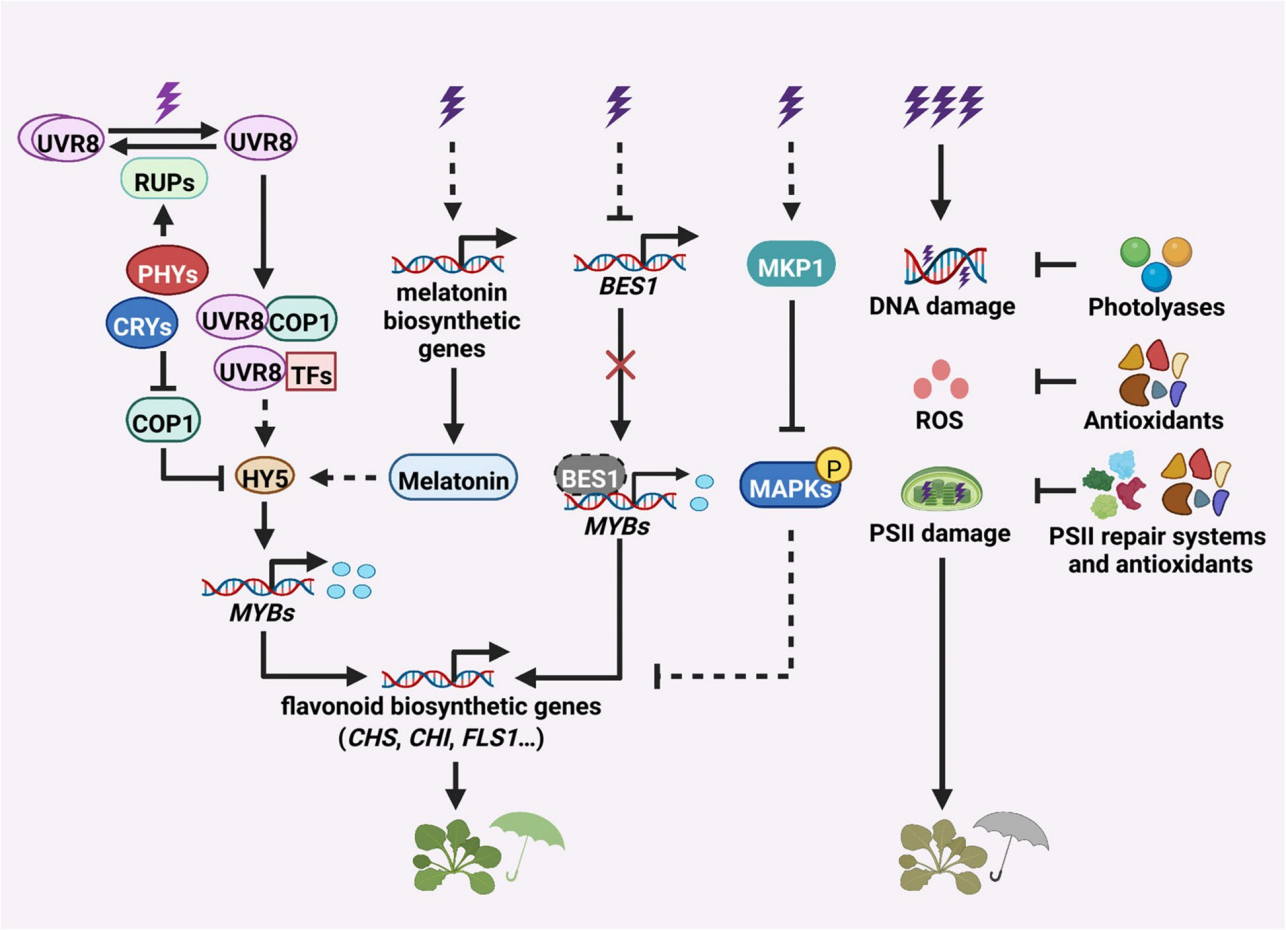
A simplified working model of UVR8-dependent and -independent regulation of UV-B stress tolerance in *Arabidopsis*. Under non-damaging UV-B radiation, UVR8 is photoactivated via a structural switch from a dimer to a monomer. Monomerized UVR8 interacts with COP1 that leads to HY5 stabilization and accumulation. HY5 associates with the promoters of *MYB* genes involved in flavonoid biosynthesis for UV-B stress tolerance. RUP1 and RUP2 negatively regulate UV-B signaling pathway through interacting directly with UVR8 to mediate UVR8 redimerization. Phytochromes and cryptochromes indirectly promote UVR8 inactivation by upregulating *RUP1* and *RUP2* expression, while they repress COP1 and stabilize HY5 to promote gene expression that confers UV-B stress tolerance. UV-B radiation upregulates the expression of melatonin biosynthetic genes. Melatonin is an antioxidant to assist UV-B stress tolerance, and also regulates UV-B signal transduction via unknown mechanism. In a UVR8-independent manner, UV-B stress downregulates *BES1* expression, and releases its inhibition of *MYBs* expression, thus promoting flavonoid accumulation and enhancing UV-B stress protection; UV-B-induced DNA damage can activate MPK3 and MPK6 downstream of their inhibitor MKP1 to negatively regulate UV-B stress tolerance. UV-B-induced DNA and PSII damage can be repaired by photolyases and PSII repair systems respectively, and ROS damage can be removed by antioxidants. Otherwise, plant survival is abolished under severe UV-B stress when UVR8-dependent and -independent machinery is insufficient for protection and repair

As described above, UV-B light can serve as an informational signal to regulate plant development, and also is a potential environmental threat to plant survival. The photoreceptor UVR8 perceives UV-B light and initiates a specific signaling pathway, to acclimate plants with the strength of UV-B stress tolerance. Alternatively, BR, MAPK and ATM/ATR signaling pathways protect plants from high-level UV-B stress independent of UVR8 (Fig. 1). Interestingly, it has also been pointed out the role of UV-B light in plant immune responses, microbial interaction, and melatonin accumulation.

Transcriptomic analyses have suggested that UV-B light co-regulates a substantial set of genes with salicylic acid (SA) and jasmonic acid (JA) which mediate pathogen defense in plants (Vandenbussche et al. 2018), further supported by species-dependent insect performance on *Arabidopsis* when exposed to UV-B radiation. In the secondary metabolites induced by UV-B light, sinapates rather than flavonoids specifically promote plant defense against the fungal pathogen *Botrytis cinerea* (Demkura and Ballare 2012). Based on the investigation of root microbiomes of *Nicotiana attenuata*, UVR8-mediated UV-B perception and response positively regulate root colonization of *Deinococcus* bacteria (Santhanam et al. 2017). Melatonin (*N*-acetyl-5-methoxytryptamine), an indolamine hormone in plants, acts as an antioxidant that plays important roles in plant defense against a variety of biotic and abiotic stresses, including UV-B stress (Hardeland and Pandi-Perumal 2005; Haskirli et al. 2020; Yao et al. 2021). In *Arabidopsis*, UV-B light facilitates the expression of melatonin biosynthetic genes such as serotonin *N*-acetyltransferase (*SNAT*), *N*-acetylserotonin methyltransferase (*ASMT*), and caffeate *O*-methyltransferase (*COMT*) (Yao et al. 2021). Melatonin treatment reduces lipid peroxidation caused by UV-B radiation and promotes UV-B responsive gene expression, indicating that melatonin not only acts as an antioxidant to affect UV-B stress tolerance, but also regulates UV-B signal transduction (Haskirli et al. 2020; Yao et al. 2021).

## Conclusion and perspectives

To date, accumulating evidence has illustrated key factors and molecular framework in plant UV-B stress tolerance (Fig. 1). UVR8 is photoactivated by UV-B light signal, and interacts with COP1 and multiple transcription factors to promote photomorphogenic development and stress acclimation. This process is regulated by phytochromes and cryptochromes as well as melatonin to optimize plant tolerance with UV-B stress. In a UVR8-independent manner, BR, MAPK and ATM/ATR signaling pathways protect plants from UV-B stress alternatively. However, there are still unsolved questions to be explored regarding the mechanism of UV-B stress acclimation and protection. For example, is there additional UV-B photoreceptor to regulate plant development and stress acclimation? Since UV-B stress inhibits the transcription of *BES1* independent of UVR8, upstream signaling factors that initiate this pathway awaits to be identified. How does UV-B signaling regulate photosynthetic performance and photoprotection? How do plants integrate UV-B stress and other biotic and abiotic stimuli? Further investigation of these and related questions will develop our understanding on plant responses to UV-B stress, and shed light on the strategy of UV-B utilization in crop production and environmental preservation.

## Data Availability

Not applicable.
